# Virtual Reality for Pediatric Postoperative Pain Management: Exploring Methods and Efficacy

**DOI:** 10.2196/68348

**Published:** 2025-08-07

**Authors:** Sidhant Kalsotra, Dillon Froass, Aneesha Gupta, Sebastian Amaya, Joseph D Tobias, Vanessa A Olbrecht

**Affiliations:** 1 Department of Anesthesiology and Pain Medicine, Nationwide Children's Hospital, Columbus, OH, United States; 2 College of Medicine, The Ohio State University, Columbus, OH, United States; 3 Department of Anesthesiology and Critical Care, Government Medical College, Jammu, India; 4 Department of Anesthesiology and Perioperative Medicine, Nemours Children’s Hospital, 1600 Rockland Road, Wilmington, DE, 19803, United States, 1 (800) 416-4441

**Keywords:** virtual reality, pediatrics, pain management, analgesia, post-operative

## Abstract

Ineffective postoperative pain management affects more than 25% of hospitalized children, leading to increased morbidity, impaired physical function, delayed recovery, prolonged opioid use, and heightened health care costs. Traditional pharmacological interventions have limitations, particularly given growing concerns over long-term opioid use in pediatric populations. Virtual reality (VR) has emerged as a promising nonpharmacological intervention for pediatric pain management, offering immersive, multisensory experiences that can effectively distract and engage patients’ attention away from pain sensations. This viewpoint examines the current evidence and prospects for VR as a component of pediatric multimodal pain management strategies. Several VR modalities have shown potential for reducing pain and anxiety in pediatric populations, including virtual reality–distraction therapy, virtual reality–exposure therapy, virtual reality–guided relaxation–based therapy, and virtual reality–biofeedback therapy. The neurobiological underpinnings of VR’s analgesic effects involve multiple mechanisms: the gate control theory explains how intense multisensory VR inputs compete with pain signal transmission, while the attention-modulation pathways involving the anterior cingulate cortex and periaqueductal gray work alongside emotional regulation pathways through amygdala connections to reduce pain perception. Recent studies involving various pediatric surgical populations have demonstrated VR’s potential to reduce postoperative pain intensity, pain unpleasantness, anxiety, and in some cases, the need for rescue analgesia. However, VR’s analgesic effects appear to be transient, typically lasting 15-30 minutes, which suggests the need for optimization in timing and frequency of VR sessions. Implementation challenges include cost considerations, technological access disparities, logistical requirements for safe use and storage, and staff training needs. As hospitals and health care systems continue to explore nonpharmacological pain management strategies, VR represents a promising adjunct to traditional approaches, potentially reducing reliance on opioid medications while improving patient experience and outcomes. Throughout this viewpoint, we address the major concepts related to VR, the use of VR in differing clinical situations, various VR-based therapy methods, and the practicality of VR to alleviate pain, as well as several key findings to date and future directions.

## Introduction

It has been estimated that more than 25% of hospitalized children have moderate to severe pain because of insufficient pain assessment and management techniques [1-4]. Ineffective postoperative pain management is associated with several negative consequences, including increased morbidity, impaired physical function and quality of life, delayed recovery, prolonged opioid use during and after hospitalization, and increased health care costs [4]. A significant amount of effort has been placed on the use of multimodal analgesia to improve these outcomes. Despite these efforts, pediatric postoperative pain remains difficult to treat, increasing the risk of persistent postoperative and chronic pain in this population [5,6]. Various studies have reported persistent pain after surgery in many different pediatric surgical populations [7-9]. Pain following surgery can persist for days to weeks, and even months, leading to time away from school, decreased quality of life, and prolonged physical and emotional disability [10,11]. Furthermore, persistent pain after surgery is a critical concern when discussing persistent opioid use after surgery [6]. This emphasizes the importance of proper pain management in pediatric patients with the optimization of postoperative analgesia and a decrease in the administration of opioid medications.

Traditional pain management therapies, including pharmacological interventions, may have limitations, particularly in children. Due to growing concerns over long-term opioid abuse among children and adolescents, alternative approaches to alleviate postoperative pain have garnered increasing interest [6,10]. In response to the opioid crisis and the need to address fear, anxiety, and the risks associated with narcotic overuse, there has been a push to identify novel and multimodal approaches to pain and anxiety control in pediatric patients undergoing surgery, aiming to reduce dependence on opioids and facilitate a rapid and effective recovery [12,13].

### Virtual Reality Modalities

Virtual reality (VR) has emerged as a promising nonpharmacological intervention for pain management in various clinical settings. VR technology immerses individuals in computer-generated environments, providing them with multisensory experiences that can distract and engage their attention, ultimately reducing their perception of pain [14]. Various types of VR technologies have been used to treat pain and anxiety, such as virtual reality–distraction therapy (VR-D), virtual reality–exposure therapy (VR-E), virtual reality–guided relaxation–based therapy (VR-GR), and virtual reality–biofeedback therapy (VR-BF). VR-D uses interactive games or simulations to redirect attention away from pain, with programs ranging from simple exploration games to complex interactive environments. VR-E creates realistic preoperative environments to familiarize patients with medical procedures, often using age-appropriate graphics and interactive elements. VR-GR incorporates meditation and breathing exercises within immersive natural environments, while VR-BF integrates physiological monitoring with virtual environments that respond to the patient’s vital signs. The selection of specific VR modalities depends on factors such as the patient’s age, procedure type, and therapeutic goals. Each of these VR modalities has been studied in various patient populations [15-19].

### VR’s Neurobiological Mechanisms

The effectiveness of VR in pain management stems from its ability to modulate pain processing through multiple neurobiological mechanisms. The fundamental mechanism involves the gate control theory of pain, where intense multisensory inputs from VR environments compete with and inhibit pain signal transmission [20]. VR operates through 2 key neural pathways: an attention-modulation pathway involving the anterior cingulate cortex and periaqueductal gray, and an emotional modulation pathway through amygdala connections that can reduce pain facilitation [21,22]. Functional neuroimaging studies have provided direct evidence for these mechanisms, demonstrating reduced activity in pain-processing brain regions during VR immersion, along with increased activation in areas associated with attention and emotional regulation [23]. This neurobiological foundation helps explain VR’s effectiveness across various clinical contexts.

In recent years, VR has gained attention as a potential tool for pain relief in pediatric patients following surgery. We conducted a comprehensive literature search to identify relevant studies on the use of VR interventions for postoperative pain management in the pediatric population. The findings of these studies are summarized in this narrative viewpoint to provide insights into the potential efficacy and benefits of VR as a nonpharmacological tool to assist in alleviating postoperative pain in children. We review the major concepts related to VR, the use of VR in various clinical situations, and the practicality of VR for pain management. Lastly, the potential future directions for VR are presented.

## Methods

### Literature Search

A systematic and comprehensive literature search was performed in the PubMed, EMBASE, PsycINFO, and CINAHL electronic databases from database inception through June 2023. The search was performed using a combination of Medical Subject Headings terms and free-text keywords. Controlled vocabulary terms (eg, “virtual reality,” “video games,” and “pediatrics”) and keywords (eg, “VR” and “postoperative pain”) were used to capture all relevant papers. Boolean operators (AND and OR) were used to refine the search. Titles and abstracts were screened for relevance by 2 independent reviewers. Full-text papers were retrieved for eligible studies. Discrepancies were resolved through discussion. A total of 8 articles were included in the final viewpoint based on relevance and quality of evidence. The search encompassed papers focusing on the use of VR in various pain therapy methods, the practicality of VR for pain alleviation, and key findings and future directions of VR use. The inclusion criteria for this viewpoint consisted of randomized clinical trials as well as observational, cohort, and case-control studies.

### Ethical Considerations

This viewpoint paper examines published literature and does not involve direct human participants’ research. The original studies cited in this viewpoint underwent their own respective ethical review processes as documented in their publications. No new patient data were collected, analyzed, or presented in this paper. No informed consent was necessary for the preparation of this viewpoint paper.

### Studies and Outcomes

#### VR-D

VR-D focuses on using VR as a tool for distraction from painful stimuli. Although the literature is mixed regarding distraction alone, using augmented distraction with VR provides an immersive and engaging experience that may be more effective than distraction alone [24-26]. By focusing their attention on the virtual environment, the perception of pain by a patient can be reduced. VR experiences can range from interactive games to calming visual and auditory simulations.

In a single-center, prospective, pilot study, Olbrecht et al [15] provided a single VR-D session to 50 children after surgery, providing them the option to play 1 of several VR games. The study included patients who had undergone several different types of procedures, all of whom required pain management by the Acute Postoperative Pain Service: 19 (38%) had abdominal surgery, 17 (34%) had Nuss repair of pectus excavatum or other chest surgery, and 14 (28%) had orthopedic procedures (such as posterior spinal fusion or major hip surgery). Before the VR session, patients completed validated questionnaires, including the Pain Catastrophizing Scale for Children and Child Anxiety Sensitivity Index, to assess whether these factors influenced VR-D effectiveness. Pain and anxiety were assessed at baseline before the VR-D session and at 3 time points after VR-D (immediately and 15 and 30 minutes after the experience). Immediately after VR-D, pain intensity decreased (median change −1.0 IQR 2.00-0, *P*<.001), remaining significant at 15 minutes (median change 0, IQR 1.00-0, *P*=.02), but not at 30 minutes. Pain unpleasantness (how much discomfort the pain was causing the patient) decreased immediately following VR-D (median −1.0, IQR 2.50-0, *P*<.001), remaining significant at both 15- and 30-minutes (*P*<.001 and *P*<.001). Finally, anxiety decreased immediately after VR-D (median 0, IQR 1.00-0, *P*<.001) and at 15 minutes (median 0, IQR 1.50-0, *P*<.001). Adjusted analyses showed significant decreases in pain intensity and unpleasantness after VR-D versus baseline. Reductions were also significant at later time points for pain unpleasantness but not intensity. No adjusted differences were seen for anxiety. Given that this study was not a randomized controlled trial (RCT), there were multiple limitations, particularly in the lack of a control group (CG). Additionally, patients received only 1 VR-D session; there was variability in the timing of the session, and a heterogeneous patient population was included. Nevertheless, this pilot study showed the feasibility of using VR-D in children after surgery and provided preliminary evidence that a single VR-D session may be associated with a reduction in pain intensity and unpleasantness in children after surgery, laying the foundation for a larger RCT to assess the efficacy and optimal timing or duration of VR for pediatric postoperative pain management.

Buyuk et al [16] assessed the effectiveness of a VR intervention following circumcision in boys aged 5 to 10 years in an RCT [16]. The study included 78 participants who were divided into a CG (n=38) and an experimental group (n=40). The CG received standard care before surgery. The experimental group received a VR intervention in addition to standard care before surgery. Two VR programs were chosen for the study: 1 simulated walking through the Amazon rainforest, and the other simulated water skiing. Before surgery, the children in the experimental group were allowed to view one of the VR programs for an average of 4.5 minutes. Data were collected using the Children’s Fear Scale (CFS), the Children’s Anxiety Meter Scale (CAM-S), and the Wong-Baker FACES Pain Rating Scale (WBS). Compared with the CG, children in the experimental group who received the VR intervention had significantly reduced CAM-S and CFS scores both before and after surgery. Furthermore, the experimental group’s postoperative WBS scores were considerably lower. The study’s greatest limitation, however, was that analgesic consumption was not consistently tracked between the groups, thereby adding significant risk for a confounding factor that would influence results. This study was notable because it was designed as an RCT and demonstrated the potential benefits of adopting a VR intervention to reduce pre- and postoperative anxiety, fear, and pain in children undergoing circumcision. Nevertheless, these limitations make it somewhat difficult to interpret the results.

Specht et al [27] conducted the first randomized study to examine the effects of VR as a nonpharmacologic intervention in the immediate postoperative period. The study used block randomization through REDCap (Research Electronic Data Capture; Vanderbilt University), with stratification by surgery type, age group (7‐12 and 13‐18 y), and gender. Patients were randomized to use either VR (n=50) using Oculus Go devices preloaded with the immersive Nature Treks VR (Greener Games) app, or an iPad (Apple Inc) tablet (control, n=56) preloaded with Child Life Specialist-approved educational games for 30 minutes after surgery in the postoperative anesthesia care unit (PACU) [27]. To maintain blinding, patients and guardians remained unaware of the alternative study arm until PACU discharge, and all participants received 5‐10 minutes of device familiarization before surgery. Assessments were carried out at multiple time points. Before surgery, patients self-reported pain using the WBS and anxiety using the Spielberger State-Trait Anxiety 6-Question Short Form (STAI) scale. Caregivers assessed their child for pain using the visual analog scale (VAS). In the PACU, pain (VAS and WBS), anxiety (STAI), and FLACC (Face, Legs, Activity, Cry, Consolability) scores were repeated before the intervention, and FLACC assessments were done at baseline and 10-, 20-, and 30-minutes during the intervention. Follow-up was done with the Post Hospitalization Behavior Questionnaire for Ambulatory Surgery at 2‐3 days and 7‐10 days after discharge. After adjusting for age, gender, and preoperative anxiety, patients using VR had significantly lower pain scores compared to the iPad group (*P*=.02). Younger patients (aged 7‐12 y) were more likely to have decreased pain scores during VR use compared to older patients (aged 13‐18 y), even after adjusting for treatment group, gender, and STAI (*P*=.04). This was most significant after 20‐30 minutes of VR use (*P*<.001). Younger patients had higher odds of withdrawal or exclusion from the study compared to older patients, after adjusting for treatment group, gender, and STAI (odds ratio 2.95, *P*=.02). There was no significant difference in opioid consumption between the VR and iPad groups (*P*=.77). Preoperative and postoperative self-reported pain scores were not significantly different between groups (*P*=.82 and *P*=.93, respectively), but at the 7‐10 day follow-up, Post Hospitalization Behavior Questionnaire for Ambulatory Surgery scores were significantly lower in the VR group (*P*=.006). Younger patients were also found to have higher odds of decreased FLACC scores over time regardless of intervention group (*P*<.05). In summary, VR was more effective than iPad use in reducing observational pain scores (FLACC), especially in younger patients, but there was no difference in opioid consumption or patient-reported pain scores between the groups. Limitations of this study included premature study termination due to COVID-19, with only 106 of a planned 150 patients included in the study. There was also a self-selection bias as this study recruited voluntary participants. There was also variability in child self-reports and the use of multiple research assistants to assess patient outcomes. Nevertheless, this RCT provides preliminary evidence that VR can reduce observational pain scores in children after surgery compared with a similar iPad distraction intervention. However, limitations such as the smaller sample size preclude definitive conclusions.

Another RCT conducted by Kumari et al [28] evaluated the effectiveness of VR as a distraction tool for children undergoing dental procedures [28]. The study included 200 participants, with 100 allocated to the immersive VR group and 100 to the nonimmersive VR group. During the immersive VR intervention, children played video games using a handheld controller, allowing them to explore and interact with the VR environment. Nonimmersive VR participants watched a cartoon movie. All participants were given a few minutes to familiarize themselves with the VR headset and controller before treatment initiation. Then a local anesthetic gel was placed, followed by a local anesthetic injection. Immediately after the local anesthetic injection, the VR equipment was retrieved from the patient, and the child’s pain perception was assessed using VAS and WBS, and anxiety was evaluated via the Faces version of the Modified Child Dental Anxiety Scale in the local language. Various dental procedures were then performed. Preoperatively, mean Modified Child Dental Anxiety Scale scores were similar between the immersive (29.20, SD 3.197) and nonimmersive (29.09, SD 3.803; *P*=.82) groups. Postoperatively, the nonimmersive group had higher Modified Child Dental Anxiety Scale scores (20.72, SD 2.822) compared to the immersive group (10.99, SD 2.227, *P*<.001). Postoperatively, the nonimmersive VR group had higher VAS pain scores (2.72, SD 0.99) compared to the immersive group (0.75, SD 0.88, *P*<.001). Similarly, postoperative WBS scores were higher in the nonimmersive group (2.78, SD 1.097) versus the immersive group (0.82, SD 1.104, *P*<.001). In summary, both the VAS and WBS pain scales indicated significantly higher postoperative pain levels in the nonimmersive versus immersive VR groups. The immersive VR environment was more effective in reducing perceived pain during intraoral injections. This study had some limitations. While the study examined immersive and nonimmersive VR effects on pain perception, it did not examine differences in the time to achieve immersion or variable effects between genders. Second, the authors did not compare pain perception based on patient education level or social status. Additionally, the bulkiness of the VR device may limit applicability for younger age groups, and future research should explore different device sizes and collaborate with manufacturers to develop more user-friendly devices suitable for all age groups.

#### About VR-E

VR-E combines VR technology with exposure therapy to mimic any triggers, stressors, or fears a patient may have, exposing the patient to these triggers or stressors in a VR environment. Such triggers may include sights, sounds, smells, and vibrations that create realistic versions of the traumatic or stressful experience. This can be particularly useful for individuals with chronic pain or phobias. Gradually exposing them to their fears in a virtual setting helps desensitize their response to pain and anxiety [29].

Eijlers et al [17] conducted a single-center, single-blinded RCT per CONSORT (Consolidated Standards of Reporting Trials) guidelines to investigate if VR-E before surgery could reduce anxiety, pain, and delirium following surgery [17]. Block randomization was performed, stratified by type of surgery: adenoidectomy or tonsillectomy, insertion of tympanostomy tubes, maxillofacial and dental procedures, or other ENT (Ear, Nose, and Throat) procedures. Assessments after randomization were performed by a blinded researcher in the holding area and during induction of anesthesia, and by blinded recovery nurses postoperatively. Participants included children aged 4 to 12 years undergoing elective maxillofacial, dental, or ENT day care surgery, with 94 randomized to VR-E versus control or care as usual (CAU, n=97). The VR environment was designed to mimic the real operating room and medical staff, familiarizing children with the surgical environment and procedures via an interactive, developmentally appropriate context using child-friendly, interactive computer-generated graphics presented via a head-mounted display for approximately 15 minutes. Two age-appropriate versions were created for children aged 4‐7 and 8‐12 years. The study assessed child anxiety at multiple time points using the modified Yale Preoperative Anxiety Scale, including at hospital admission (VR-E median 26.7 vs CAU 26.7, *P*=.70), in the holding area (VR-E 28.3 vs CAU 28.3, *P*=.765), and during the induction of anesthesia (VR-E 40.0 vs CAU 38.3, *P*=.86). Self-reported anxiety on VAS was also measured at admission (VR-E 3.0 vs CAU 1.5, *P*=.41), in the holding area (VR-E 3.0 vs CAU 3.5, *P*=.75), and postoperatively (*P*>.05 at all postoperative time points). Pain was assessed in the recovery room through child self-report on the Faces Pain Scale-Revised (VR-E 2.0 vs CAU 2.0, *P*=.70), nurse observation with the FLACC scale (VR-E 0.0 vs CAU 0.0, *P*=.67), and parent report on the Parents’ Postoperative Pain Measure (VR-E 3.0 vs CAU 3.0, *P*=.41). Emergence delirium was measured by the Pediatric Anesthesia Emergence Delirium Scale and was not significantly different between groups (*P*=.27). The only significant finding was that after tonsillectomy, the VR-E group required less postoperative rescue analgesia (55%) compared to controls (95.7%; *P*=.002). In the study, VR-E did not significantly reduce postoperative anxiety, postoperative pain, or emergence delirium compared to standard care but was associated with a reduction in postoperative rescue analgesia after tonsillectomy. The strengths of the study included a large sample size, limited missing data, standardized assessment tools, blinding of participating staff, and a narrow range of surgical procedures. However, the study also had limitations, including the use of only 1 postoperative assessment, the lack of measurement of satisfaction with VR-E, a large number of children discontinuing study participation (n=21), and the exclusion of the most anxious children in the study population.

Yaz and Yilmaz [14] conducted an RCT following CONSORT guidelines, with 132 children block randomized into 3 equal groups: educational animation VR (n=44), documentary VR (n=44), and control (n=44). The study used validated assessment tools, including the CFS and WBS, administered through an information form [14]. A short, animated video was shown to the educational animation virtual reality group (AG) to educate participants on preoperative and postoperative procedures. It covered the step-by-step process from the child’s initial hospital visit for examination and vital sign checks, wearing a surgical gown, going to the operating room, and postoperative care, including pain assessment. The documentary virtual reality group (DG) saw a short documentary video with instrumental music that included forests, trees, and flowers. The documentary film was chosen by the researchers and consultants based on the cognitive levels of the children. The CG received routine care [14]. At baseline, there were no significant differences between the 3 groups in mean self-reported (AG 2.89, SD 0.72, DG 2.82, SD 0.58, CG 2.98, SD 0.66; *P*<.001) and parent-reported fear scores (AG 2.77, SD 0.71, DG 2.52, SD 0.62, CG 2.68, SD 0.67; *P*<.001). After watching the video, the animation group had significantly decreased self- and parent-reported mean fear scores (decrease from 2.89, SD 0.72 to 0.91, SD 0.85 and 2.77, SD 0.71 to 1.3, SD 0.76, respectively; *P*<.001). The differences in the change in fear scores between pre- and postvideo were significantly greater for the educational animation group compared to the documentary (*P*<.001 for child and parent ratings) and CGs (*P*<.001 for child and parent ratings). Intergroup comparison of mean pain scores showed an overall difference between groups (*P*<.001). Based on children’s self-reports, pain scores were lower in the animation group compared to the control and documentary groups, with no difference between the documentary and CGs (*P*=.10). However, both parent and nurse evaluations of children’s pain found that scores were lower in the animation versus documentary and CGs. Additionally, parents and nurses reported a statistically significant difference in pain scores between documentary and CGs (*P*=.035 for parents, *P*=.03 for nurses), unlike the children’s self-reports. In the study, VR-E was effective in reducing preoperative fear and postoperative pain. While this intervention shows promise, larger studies are needed to better assess the value of this therapy.

#### About VR-GR

Combining strategies of traditional mind-body therapies, such as relaxation, slow breathing, mindfulness, and biofeedback, with the immersive nature of VR opens new possibilities for multimodal analgesia and has the potential to simultaneously minimize acute postoperative pain and opioid consumption. VR-GR is a promising mechanism to deliver mind-body–based therapy (guided relaxation) and potentially enhance the effectiveness of these techniques to promote pain relief [30].

Olbrecht et al [18] provided a single 10-minute VR-GR session to 51 children and adolescents after surgery using the Mindfulness Aurora (Stanford CHARIOT [Childhood Anxiety Reduction through Innovation and Technology]) app, an app that provides a guided relaxation session. Patients were followed by the Acute Pain Service and completed the Pain Catastrophizing Scale for Children and Child Anxiety Sensitivity Index before the intervention. Using the Mindfulness Aurora app, which provides a guided relaxation session, the study assessed changes in pain intensity (primary outcome) and pain unpleasantness and anxiety (secondary outcomes) at immediate, 15-minute, and 30-minute postintervention timepoints. Pain intensity decreased immediately (*P*<.001) and at 30 minutes (*P*=.04) following the session, but not at 15 minutes (*P*=.16) after VR-GR compared to baseline. Pain unpleasantness decreased at all evaluated time points (*P*<.001). Anxiety decreased immediately (*P*=.02) but not at 15- or 30-minutes after VR-GR. Patients with higher anxiety sensitivity scores had greater reductions in pain intensity (*P*=.04) and unpleasantness (*P*=.01). This study also assessed patient and parent satisfaction with VR. Overall, 96% of children would recommend VR, and 88% believed they felt calmer and could better tolerate pain after VR-GR. Parents reported similarly positive experiences. While this is the first study to combine VR with mind-body therapy for pediatric postoperative pain, limitations of this study included the lack of a CG, potential interaction effects with the study team, no data on analgesic use, variable timing of the postoperative visit, and potential bias in self-reported outcomes. Additionally, given that the study was not a randomized clinical trial, no causative relationships between VR-GR and effects on pain and anxiety can be drawn. However, this pilot study provides initial evidence that a single VR-GR session is associated with a reduction in pain intensity and unpleasantness in children after surgery. These effects were immediate and sustained for up to 30 minutes after the experience. This study also lays the foundation for and highlights the need for an RCT assessing the use of VR-GR to reduce pain, anxiety, and opioid consumption in children and adolescents having surgery.

#### About VR-BF

More recently, VR-BF has emerged as a potentially novel method to reduce pain, anxiety, and opioid consumption in children after surgery. Biofeedback, a nonpharmacological, complementary therapy, teaches patients skills necessary for behavioral adjustments that affect involuntary systems. Slow breathing, for example, increases heart rate variability, which reduces pain by downregulation of the sympathetic nervous system [19]. VR-BF is a technique that uses VR to deliver biofeedback, thereby increasing the immersion of this therapy. To date, no randomized clinical trial has been performed using VR-BF for pain and anxiety management, and, as such, it remains unclear how this therapy needs to be integrated into perioperative management.

Orgil et al [31] propose a single-center, randomized pilot trial with 70 patients, aged 12‐18 years, undergoing surgery expected to cause moderate-severe pain requiring at least 1 night of hospitalization. Participants will be randomized to VR-BF using the ForeVR (Biofeedback VRx, Inc) VR platform for breathing exercises (n=35) or a CG using the Manage My Pain (ManagingLife), a commercially available pain management app (n=35) [31]. VR-BF will be used preoperatively for education and training, and postoperatively for pain management. The primary outcome focuses on feasibility metrics, including recruitment, enrollment, retention, and protocol adherence, while secondary outcomes assess VR-BF acceptability and pilot efficacy measures, including pain, anxiety, and opioid consumption. Participants will document session usage and pain and anxiety measures using the numerical rating scale. Some limitations of this planned study include limits in generalizability, both based on the specific patient population used as well as the academic health care setting. Nevertheless, if the feasibility of this intervention is demonstrated, a larger clinical efficacy trial will then be warranted.

#### Virtual Reality Cognitive Behavioral Therapy

VR can also be used as a tool in conjunction with cognitive behavioral techniques to manage pain. By combining VR environments with cognitive strategies, individuals can learn to identify and challenge negative thoughts and emotions associated with pain. This approach modifies the perception and interpretation of pain, leading to reduced suffering [32]. Virtual reality cognitive behavioral therapy combines many of the previously mentioned principles of other VR strategies that help the patient focus on biofeedback (breathing pattern), while also providing distraction and immersive enjoyment. The US Food and Drug Administration recently approved the use of a virtual reality cognitive behavioral therapy device for use in adult patients with diagnosed chronic back pain; however, the use of this technique in pediatric patients has not yet been explored.

### Implementation Challenges and Clinical Integration

While VR shows promise for pediatric pain management, there are also potential barriers to the widespread implementation of VR devices, such as cost, access to technology, and staff training requirements. These challenges can significantly impact the adoption and sustainability of VR programs in health care settings and must be addressed for successful clinical implementation.

Financial considerations present a significant initial barrier. Initial costs include purchasing VR headsets, software licenses, and the necessary infrastructure to support the technology [33]. For many health care systems, particularly those in underserved areas, these expenses can be prohibitive [34]. Additionally, ongoing operating expenses, including maintenance and software updates, and replacement of worn components must be considered as well [33,35]. Access to VR technology poses another barrier, that is, disparities in technology availability can exacerbate existing inequalities in patient care. Hospitals in urban areas may be more likely to secure funding for advanced technologies such as VR compared to those in rural or underserved urban settings [35]. These inequities in access may limit the potential benefits of VR and hinder universal integration into pediatric pain management and therapeutic programs. In the health care facilities that are able to implement VR, additional logistical challenges must be considered, such as the need for dedicated spaces where the technology can be used safely and effectively, as well as dedicated spaces for proper storage of VR devices while maintaining proper infection control protocols. Inadequate space or resources to support these conditions further complicates the availability and use of VR [33,36].

Lastly, staff training represents another crucial consideration for successful VR implementation. Proficiency in VR equipment setup, maintenance, and troubleshooting is necessary to ensure proper patient use [37]. This training must be integrated into existing staff education programs while minimizing disruption to clinical care. Clear protocols must be established for documenting VR usage in medical records and coordinating care between team members. Health care organizations must carefully consider these financial, technical, and training challenges when developing implementation strategies to ensure successful and sustainable VR program integration into clinical practice.

## Discussion

### Principal Findings

This viewpoint highlights VR’s potential as a complementary tool for pediatric perioperative pain, with evidence supporting short-term reductions in pain and anxiety—particularly in anxious or younger patients. However, effects are often transient (15‐30 min) and limited by variability in protocols, hardware challenges, and a lack of standardized dosing. While promising, larger studies are needed to optimize VR integration, including repeated sessions, objective pain measures, and cost-effective implementation tailored to the surgical context and developmental stage. Collaboration between clinicians and developers will be critical to advance this field.

In this narrative viewpoint, we analyzed 8 studies using VR as an intervention for perioperative pain and anxiety management in pediatric patients. Out of these, 6 were RCTs and 2 were prospective pilot studies. The studies investigated various VR modalities, including VR-D, VR-GR, and VR-E. Two prospective pilot studies from Olbrecht et al demonstrated promising preliminary results [15,18]. In their VR-D study, they found that there was an immediate decrease in pain intensity that lasted up to 15 minutes postintervention, as well as pain unpleasantness reduction that persisted for 30 minutes. The VR-GR trial found similar transient reductions in pain intensity and anxiety, but these were not clinically significant (defined as a reduction of ≥2 points on the numerical rating scale or a 30% reduction in pain). The transient nature of VR’s analgesic effects warrants careful consideration in future research and clinical applications. The finding that pain relief diminishes within 15‐30 minutes postintervention suggests that the mechanisms of VR-mediated pain relief may be primarily active during the immersive experience. Several factors could influence the duration of pain relief, including the type of VR content (distraction vs guided relaxation), level of patient engagement, and timing of the intervention relative to pain peaks. The observation that patients with higher anxiety sensitivity scores experienced greater pain reduction suggests that patient characteristics may also play a role in the sustainability of VR’s effects. Understanding these factors is crucial for optimizing VR interventions and potentially extending their therapeutic benefits.

Table 1 presents a comprehensive overview of the 8 published studies investigating VR interventions for perioperative pain management in pediatric populations discussed in this viewpoint. The study designs include 6 RCTs and 2 prospective observational pilot studies, with sample sizes ranging from 50 to 200 participants. The patient populations encompass children and adolescents aged 4‐21 years undergoing various procedures, including major surgeries (abdominal, chest, or orthopedic), minor procedures (circumcision or dental interventions), and day surgeries (ENT or maxillofacial). Four distinct VR modalities are represented: VR-D, VR-E, VR-GR, and VR-BF. Intervention timing varied across studies, with applications occurring preoperatively, immediately postoperatively in PACU, or during later recovery phases. Primary outcomes assessed include pain intensity and unpleasantness using validated pediatric measures (numerical rating scale, VAS, WBS, or FLACC), anxiety scores (STAI, CFS, or CAM-S), and analgesic consumption. Key methodological limitations are noted for each study, highlighting challenges in the current evidence base, including heterogeneous interventions, variable assessment timing, and inconsistent control conditions. These studies collectively demonstrate preliminary evidence for VR’s potential in reducing pediatric perioperative pain and anxiety, with effects that appear time-limited and potentially influenced by patient characteristics such as age and baseline anxiety.

**Table 1.** Characteristics and findings of VR^a^ studies for pediatric perioperative pain management.

Study	Design	Population or sample size	VR modality	Key findings	Limitations
Olbrecht et al [15]	Single-center, prospective pilot study	50 children postsurgery (38% abdominal, 34% chest, and 28% orthopedic); ages 7‐21 years	VR-D^b^ with choice of games	Pain intensity decreased immediately (*P*<.001) and at 15 min (*P*=.02); pain unpleasantness decreased up to 30 min (*P*<.001); anxiety decreased up to 15 min (*P*<.001)	No control group; single VR session; variable timing of sessions; heterogeneous population; no analgesic use data
Buyuk et al [16]	RCT^c^	78 boys undergoing circumcision (40 VR and 38 control); ages 5‐10 years	VR-D (Amazon rainforest or water skiing simulation)	Reduced CAM-S^d^ and CFS^e^ scores pre- or postsurgery; lower postoperative WBS^f^ scores in VR group	Analgesic consumption not tracked; limited pain score measurements; only parent and researcher evaluations
Specht et al [27]	RCT	106 patients (50 VR and 56 iPad); ages 7‐18 years	VR-D using Nature Treks VR	Lower FLACC^g^ scores with VR vs iPad (*P*=.02); better response in younger patients (7‐12 y); no difference in opioid consumption	Premature termination (COVID-19); self-selection bias; multiple assessors
Kumari et al [28]	RCT	200 children (100 immersive VR and 100 nonimmersive); ages 6‐12 years	VR-D during dental procedures	Lower pain with immersive VR (VAS^h^ 0.75, SD 0.88 vs 2.72, SD 0.99); reduced anxiety in both groups	Device size limitations; no analysis of immersion time
Eijlers et al [17]	Single-center, single-blinded RCT	191 children (94 VR-E^i^ and 97 control); ages 4‐12 years	VR-E mimicking the OR^j^ environment	No effect on anxiety, pain, or delirium; reduced rescue analgesia after tonsillectomy (55% vs 95.7%, *P*=.002)	Single postoperative assessment; high discontinuation rate (n=21); excluded most anxious patients
Yaz and Yilmaz [14]	RCT	132 children (44 educational VR, 44 documentary VR, and 44 control); ages 6‐12 years	VR-E and VR-D	Lower pain scores in animation group; significant reductions in preoperative fear	Limited assessment timepoints; further validation needed
Olbrecht et al [18]	Pilot observational study	51 children postsurgery; ages 7‐21 years	VR-GR^k^ using Mindfulness Aurora app	Pain intensity decreased immediately (*P*<.001) and at 30 min (*P*=.04); pain unpleasantness decreased at all time points; anxiety decreased immediately (*P*=.02)	No control group; variable timing; no analgesic data; single session
Orgil et al [31]	Proposed randomized pilot trial	70 adolescents (35 VR-BF^l^ and 35 control); ages 12‐18 years	VR-BF	Study ongoing; primary outcome: feasibility of VR-BF integration	Limited generalizability; specific age group; academic setting

a VR: virtual reality.

b VR-D: virtual reality–distraction therapy.

c RCT: randomized controlled trial.

d CAM-S: Children’s Anxiety Meter Scale.

e CFS: Children’s Fear Scale.

f WBS: Wong-Baker FACES Pain Rating Scale.

g FLACC: Face, Legs, Activity, Cry, Consolability.

h VAS: visual analog scale.

i VR-E: virtual reality–exposure therapy.

j OR: operating room.

k VR-GR: virtual reality–guided relaxation–based therapy.

l VR-BF: virtual reality–biofeedback therapy.

Among the RCTs, Buyuk et al [16] found that patients who underwent circumcision and used VR had significant reduction in anxiety and fear scores both pre- and postoperatively, with reduced postoperative pain scores as measured by the WBS. Specht et al [27] demonstrated VR’s superiority for immediate postoperative pain management over iPad use, with notably better outcomes in younger patients despite a higher withdrawal rate. Kumari et al [28] specifically assessed immersive versus nonimmersive VR during dental procedures, finding both methods effective for anxiety reduction but superior pain control with immersive VR during intraoral injections. Eijlers et al [17] conducted a larger trial to investigate the VR exposure before elective day care surgery. While they found no significant impact on anxiety or emergence delirium, they did observe a clinically important reduction in rescue analgesia following more painful surgeries. Yaz and Yilmaz [14] found that educational animated VR movies effectively reduced both preoperative fear and postoperative pain scores compared to CGs. The ongoing trial by Orgil et al is investigating the feasibility of perioperative biofeedback-based VR integration.

### Limitations and Generalizability

Several important limitations were noted across these studies. First, the lack of standardization in VR session timing and duration makes comparisons across studies challenging. Future studies should establish protocolized VR dosing schedules. Second, potential self-reporting bias in pain assessments may affect reliability, particularly in younger children. While some studies (eg, Buyuk et al [16]) used parent or researcher observations, future trials could integrate objective biomarkers (eg, heart rate variability) to complement subjective measures. Third, varying CG designs (eg, standard care vs active comparators such as iPads) impact interpretation of results. Meta-analyses should account for these differences through subgroup analyses. Fourth, studies by Olbrecht et al [15,18] did not evaluate changes in opioid consumption or control for pain medication usage, potentially confounding pain score reductions. Their VR sessions were also single sessions with irregular timing, limiting generalizability. Future work should track analgesics systematically and test repeated VR sessions. Fifth, in the study by Buyuk et al [16], only parents and researchers evaluated pain score measurements, introducing potential observer bias. Blinded assessors could mitigate this in future trials. Sixth, the study by Specht et al [27] was ended prematurely because of the COVID-19 pandemic, had no power estimates, and might have been biased by self-selection. These issues likely reduced statistical robustness. Replication with larger, randomized samples is needed. Seventh, the study by Eijlers et al [17] excluded the most anxious patients (those receiving anxiolytic premedication), potentially underestimating VR’s effects in high-anxiety populations. They also reported that 21 children (particularly ages 4‐5 years) discontinued due to bulky headsets. Newer, child-specific VR hardware may improve adherence. Finally, the study by Orgil et al [31] noted that academic hospital settings and specific demographics limited generalizability. Community-based trials are warranted to assess real-world applicability.

### Major Versus Minor Surgeries

While this narrative viewpoint encompassed studies looking at a wide variety of types of surgeries, further analysis into the efficacy of VR interventions on the type of surgery could help provide clearer guidelines for patient selection for VR use for pain management. A systematic review and meta-analysis by Ding et al [38] classified studies, mostly using VR-D, into major and minor surgery groups based on prior literature, finding that VR was able to reduce pain after both major and minor surgeries. However, the specific efficacy of pain relief may vary, since major surgery puts a greater degree of pain stimulus on patients, resulting in a higher degree of pain intensity [38]. Thus, the type of surgery is an important consideration in whether a patient should be offered VR therapy for pain management, as its efficacy can vary based on baseline pain.

### Long-Term Efficacy and Follow-Up

While the current evidence suggests VR as a promising nonpharmacological intervention for perioperative pain management in pediatrics, the transient nature of its analgesic effects remains a significant consideration. The feasibility of repeated VR sessions in the pediatric perioperative period has not yet been explored; the feasibility of repeated VR sessions in other settings has been assessed. An RCT performed by McConnell et al [39] explored the feasibility of incorporating VR-GR into a physical therapy program for chronic back pain [39]. Patients in the VR group engaged in 12 sessions, 21 minutes each, over 6 weeks. Results of this study suggest that repeated VR sessions over multiple weeks may be acceptable and feasible. Another study by Shaw et al [40] involved eight 35-minute interactive, VR-D sessions over 4 weeks as an intervention for chronic pain in patients with multiple sclerosis. This study concluded that repeated VR sessions are feasible [40]. While these studies are not specific to pediatric perioperative pain management, they do suggest that repeated VR sessions are feasible; however, studies specifically addressing repeated VR use in the perioperative period are warranted to understand optimal timing and frequency of interventions for maximizing therapeutic value in this unique clinical context.

### Comparison With Other Nonpharmacological Methods

VR offers unique advantages compared to traditional nonpharmacological pain management approaches. Unlike music therapy, which primarily provides passive distraction, VR creates an immersive environment that actively engages multiple sensory systems [41]. While cognitive behavioral therapy requires multiple sessions to develop coping strategies, VR can provide immediate pain relief through distraction and may be more engaging for pediatric patients [42]. However, VR’s higher cost and technical requirements may limit its accessibility compared to simpler interventions such as music therapy or guided imagery. These comparisons highlight VR’s potential role as a complementary tool within a comprehensive pain management strategy rather than a replacement for existing approaches.

### Future Directions

Future research should monitor analgesic consumption and integrate objective pain assessments while evaluating cost-effectiveness and implementation strategies in clinical settings. Future developments should focus on creating standardized protocols for different surgical procedures and age groups, developing more ergonomic and child-friendly VR devices, and establishing clear guidelines for optimal timing and duration of VR interventions. Collaboration between health care providers, VR developers, and researchers will be essential to create age-appropriate content and address current technical limitations. Additionally, larger multicenter trials are needed to validate these findings across diverse patient populations and clinical settings. The collective findings from these studies provide a foundation for future research while highlighting the potential use of VR technology in pediatric pain management.

## Abbreviations:

AG: animation virtual reality group
CAM-S: Children’s Anxiety Meter Scale
CAU: care as usual
CFS: Children’s Fear Scale
CG: control group
CHARIOT: Childhood Anxiety Reduction Through Innovation and Technology
CONSORT: Consolidated Standards of Reporting Trials
DG: documentary virtual reality group
ENT: Ear, Nose, and Throat
FLACC: Face, Legs, Activity, Cry, Consolability
PACU: postoperative anesthesia care unit
RCT: randomized controlled trial
REDCap: Research Electronic Data Capture
STAI: State Trait Anxiety 6-question short form
VAS: visual analog scale
VR: virtual reality
VR-BF: virtual reality–biofeedback therapy
VR-D: virtual reality–distraction therapy
VR-E: virtual reality–exposure therapy
VR-GR: virtual reality–guided relaxation–based therapy
WBS: Wong-Baker FACES Pain Rating Scale
